# Exopolysaccharides from the Green Microalga Strain *Coelastrella* sp. BGV—Isolation, Characterization, and Assessment of Anticancer Potential

**DOI:** 10.3390/cimb46090614

**Published:** 2024-09-16

**Authors:** Tanya Toshkova-Yotova, Inna Sulikovska, Vera Djeliova, Zdravka Petrova, Manol Ognyanov, Petko Denev, Reneta Toshkova, Ani Georgieva

**Affiliations:** 1 Department of Plant Ecophysiology, Institute of Plant Physiology and Genetics, Bulgarian Academy of Sciences, Acad. G. Bonchev Str., 21, 1113 Sofia, Bulgaria; t_toshkova_yotova@abv.bg; 2 Department of Pathology, Institute of Experimental Morphology, Pathology and Anthropology with Museum, Bulgarian Academy of Sciences, Acad. G. Bonchev Str., 25, 1113 Sofia, Bulgaria; inna_sulikovska@ukr.net (I.S.); zdr.z1971@abv.bg (Z.P.); reneta.toshkova@gmail.com (R.T.); 3 Department of Molecular Biology of Cell Cycle, Institute of Molecular Biology “Acad. R. Tsanev”, Bulgarian Academy of Sciences, Acad. G. Bonchev Str., 21, 1113 Sofia, Bulgaria; vera@bio21.bas.bg; 4 Laboratory of Biologically Active Substances, Institute of Organic Chemistry with Centre of Phytochemistry, Bulgarian Academy of Sciences, 139 Ruski Blvd., 4000 Plovdiv, Bulgaria; manol.ognyanov@orgchm.bas.bg (M.O.); petko.denev@orgchm.bas.bg (P.D.)

**Keywords:** *Coelastrella* sp. BGV, antitumor activity, cell viability, proliferation, migration, apoptosis

## Abstract

Algal metabolites have been extensively studied as potential anticancer therapeutics. Among them, polysaccharides have attracted much attention because of their beneficial biological effects and safety. In the present research, the chemical characteristics, antitumor, and proapoptotic activities of extracellular polysaccharides (EPS) isolated from a new Bulgarian strain of the green microalga *Coelastrella* sp. BGV were investigated. A fast and convenient method of precipitation with cold ethanol was used to isolate EPS from the culture medium. The chemical characteristics of the isolated EPS were examined by colorimetric and spectrophotometric analyses, HPSEC-RID and HPLC-UV chromatography, and FT-IR spectroscopy. The results showed that the isolated EPS sample consists of three carbohydrate fractions with different molecular weights (11.5 × 10^4^ Da, 30.7 × 10^4^ Da, and 72.4 × 10^4^ Da, respectively) and contains 7.14 (*w*/*w*%) protein. HPLC-UV analysis revealed the presence of galactose and fucose. The total uronic acid content in the sample was 4.5 (*w*/*w*%). The IR-FT spectrum of EPS revealed the presence of various functional groups typical of a polysaccharide (or proteoglycan) composed primarily of neutral sugars. The anticancer potential of the obtained EPS was assessed using cell lines with cancerous and non-cancerous origins as in vitro experimental models. The results of the performed MTT assay showed that EPS reduced the viability of the cervical and mammary carcinoma cell lines HeLa and MCF-7, while the control non-cancer cell lines BALB/3T3 and HaCaT were less affected. The HeLa cell line showed the highest sensitivity to the effects of EPS and was therefore used for further studies of its anticancer potential. The ability of EPS to inhibit cancer cell migration was demonstrated by wound-healing (scratch) assay. The cell cycle FACS analysis indicated that the EPS treatment induced significant increases in the sub G1 cell population and decreases of the percentages of cells in the G1, S, and G2-M phases, compared to the control. The fluorescent microscopy studies performed using three different staining methods in combination with Annexin V-FITC flow cytometric analysis clearly demonstrate the ability of EPS to induce cancer cell death via the apoptosis pathway. Moreover, an altered pattern and intensity of the immunocytochemical staining for the apoptosis- and proliferation-related proteins p53, bcl2, and Ki67 was detected in EPS-treated HeLa cancer cells as compared to the untreated controls. The obtained results characterize the new local strain of green microalgae *Coelastrella* sp. BGV as a producer of EPS with selective antitumor activity and provide an opportunity for further studies of its pharmacological and biotechnological potential.

## 1. Introduction

Cancer is the second leading cause of mortality worldwide, and more effective therapies to control these severe diseases are urgently needed. A distinctive feature of cancer is the uncontrolled proliferation and spread of tumor cells and the loss of apoptosis. Compounds that block or suppress the proliferation of tumor cells by inducing apoptosis are considered to have potential as anticancer drugs [1]. The interest in algae as one of the major natural sources of novel bioactive compounds with health-promoting immunostimulating and anticancer properties has intensified in recent years. In general, microalgae are rich in a spectrum of compounds including, high-quality proteins (phycobiliproteins, enzymes and oligopeptides), polysaccharides (agar, carrageenan, alginates, fucoidan, etc.), long-chain polyunsaturated fatty acids (especially ω-3 and ω-6 fatty acids), pigments (carotenoids: β-carotene, lutein, astaxanthin, fucoxanthin, etc. and phycocyanin), sterols (sitosterol, fucosterol, etc.), polyphenols, vitamins B12, C, and E, biogenic elements (Ca, P, Na, K, etc.), dietary fiber, etc. [2,3,4].

Among the high-value compounds from microalgae, EPS are very promising. Polysaccharides are natural biopolymers, composed of monosaccharides (hexoses and pentoses) covalently linked by glycosidic bonds in linear or branched chains with a great variety of molecular weights, monosaccharide compositions, and structural conformations. Based on their function, polysaccharides can be divided into three categories: (i) structural polysaccharides that build cell walls, (ii) storage (reserve) polysaccharides that accumulate inside the cell, and (iii) matrix (protective) polysaccharides released into the environment. The latter are subdivided into two different classes—capsular, which remain attached to the cell wall, and free, which are entirely released into the environment and are described as extracellular polysaccharides [5,6]. The best producers of exopolysaccharides were found in different species of microalgae from *Cyanophyta* and *Rhodophyta*, as well as in some types from *Chlorophyta* and *Dinophyta* [7,8]. The polysaccharide content, composition, and structure are highly variable depending on numerous influencing factors, such as algal species and strain, the area of cultivation, seasonal, physiological, and environmental variations, cultivation parameters, and culture age. Variations can even occur between samples prepared in the same way from different batches of a given species of algae. The type and conditions of extraction also have a very decisive influence on the polysaccharide content [9,10,11].

The microalgae polysaccharides attract scientific attention due to their diverse biological activities, including the anticoagulant, antithrombotic, immunomodulatory, antiviral, antibacterial, hypoglycemic, antimutagenic, radioprotective, anti-oxidative, antiulcer, anticancer, and anti-inflammatory effects, and find wide application in the pharmaceutical, environmental, cosmetics, and food industries (including functional foods, feed, nutritional supplements, colorants, and drinks) [6,7,8,11,12,13,14,15,16].

Numerous studies have indicated that polysaccharides from microalgae sources exhibit antitumor efficacy by inducing apoptosis, inhibiting proliferation and angiogenesis, and modulating the immune response in different cancer models [7,15,17,18,19,20,21].

At present, there are no reports about the pharmacological action of the extracellular polysaccharides from *Coelastarella* species. As a part of our effort to explore the therapeutic potential of the local microalgae strains, we undertook a study on the anticancer activity of the metabolites isolated from the green microalga *Coelastrella* sp. BGV. Our previous studies have shown that aqueous and oil extracts and fatty acids from this strain inhibited cell proliferation and induced apoptotic changes in HeLa tumor cells [22,23,24]. In this study, we isolated extracellular polysaccharides (EPS) from the green microalga *Coelastrella* sp. BGV, investigated its chemical characteristics, and assessed the antitumor and proapoptotic effects on HeLa human cervical cancer cells. The results obtained provide new data regarding the mechanism of action of EPS against cervical cancer and demonstrate the potential for developing EPS as an agent for cervical cancer prevention and/or therapy. However, more extensive studies are required to better understand the biological mechanisms underlying the antitumor effect of *Coelastrella* EPS and to validate its potential for applications in cancer therapy.

## 2. Materials and Methods

### 2.1. Materials and Chemicals

*Coelastrella* sp. BGV was obtained from the Algae Culture Collection of the Institute of Plant Physiology and Genetics at the Bulgarian Academy of Sciences.

Monosaccharides (L-rhamnose, D-arabinose, D-xylose, D-mannose, D-glucose, D-galactose, and D-fructose) were purchased from the Sigma-Aldrich Chemical Company (St. Louis, MO, US). Dulbecco’s Modified Eagle Medium (DMEM), fetal bovine serum, antibiotic solution (penicillin–streptomycin), phosphate-buffered solution (PBS) and trypsin–EDTA solution (2.5 g/L trypsin and 0.2 g/L EDTA), 3-[4,5-dimethylthiazol-2-yl]-2,3-diphenyl tetrazolium bromide (MTT), Acridine Orange (AO), and ethidium bromide (EtBr), were purchased from Merck (Darmstadt, Germany). The Annexin V Apoptosis Detection Kit: sc-4252 AK was the product of Santa Cruz Biotechnology, Inc., Dallas, TX, USA.

### 2.2. Cell Lines and Culture Conditions

The human cancer cell line HeLa (CCL-2); MCF-7 (HTB-22) and mouse fibroblast cell line BALB/3T3 (CCL-163) were purchased from the American Type Culture Collection (ATCC). The non-tumor human keratinocyte cell line HaCaT was obtained from the CLS Cell Lines Service (Eppelheim, Germany). Cells were maintained in Dulbecco’s modified Eagle’s medium (DMEM) supplemented with 10% heat-inactivated fetal bovine serum, 2 mM L-glutamine, 50 U/mL penicillin, and 50 mg/mL streptomycin. Both cell lines were cultivated at 37 °C in a humidified atmosphere with 5% CO_2_. After achieving 60–80% confluency, the cells were trypsinized with 0.25% trypsin (dissolved in PBS, pH 7.4), counted, and placed at the necessary density for each experiment.

### 2.3. Algal Strain and Growth Conditions

The green microalga *Coelastrella* sp. strain BGV was isolated from stagnant water in a metal trough near the village of Varvara, Bulgaria (N 42° 10′; E 24° 0–7′) [25]. The strain is maintained in the algal culture collection of the Laboratory of Experimental Algology, at the Institute of Plant Physiology and Genetics at the Bulgarian Academy of Sciences. Algae were cultured as monospecific non-axenic cultures in glass bottles containing 200 mL of autoclaved Šetlik medium modified by Georgiev et al. [26] at 28 °C on a block for intense cultivation. Other experimental conditions included continuous unilateral illumination with cool-white fluorescent lamps at a photon flux density of 132 μmol m^−2^ s^−1^ and a carbon source provided by bubbling 2–3% CO_2_ (*v*/*v*) in air through the suspensions. The cultures were hand-shaken daily.

### 2.4. Extraction of Extracellular Polysaccharide

The extracellular polysaccharides from *Coelastrella* sp. BGV were isolated and purified from the cell-free culture liquids using previously described methods [27]. In brief, cultures grown for 20 days under the optimized conditions were subjected to centrifugation at 5000× *g* for 30 min to obtain culture filtrates containing both the released EPS and the culture medium. The resulting supernatant (cell-free culture liquid) was precipitated with cooled 99% ethanol in a ratio of 1:2 (*v*/*v*) and pelleted at 5000× *g* for 20 min at room temperature. The precipitate was rewashed three times with 65% ethanol to remove any contaminants (lipophilic substances, carotenoid and chlorophyll-related pigments, and other low molecular weight compounds), and dried at 37 °C. The precipitated materials were dissolved in sterile double distilled water (1%, *w*/*v*) on a magnetic stirrer and then extensively dialyzed against distilled water using a cellulose membrane dialysis tubing (Sigma, St. Louis, MO, USA) and finally, were freeze-dried and lyophilized. The EPS was preserved at 4 °C. The obtained crude polysaccharide was used for chemical analysis and bioassays.

### 2.5. Characterization of Extracellular Polysaccharide (EPS)

#### 2.5.1. Chemical Composition Analysis

The total amount of carbohydrates in EPS was estimated using the phenol–sulfuric acid method of Dubois et al. [28], in which glucose was used as a standard. The protein content of the polysaccharide sample was determined using the dye-binding method of Bradford using bovine serum albumin as a standard [29]. The total uronic acid content of the EPS was analyzed colorimetrically with 3-hydroxybiphenyl by the method of Blumenkrantz and Asboe-Hansen [30], using D-galacturonic acid (D-GalA) as a standard (5.0–100.0 μg/mL) (Sigma-Aldrich Chemical Co., St. Louis, MO, USA). The acetyl content was determined photometrically using the hydroxamic acid reaction method of McComb and McCready [31], using β-D-glucose-pentaacetate (24–120 μg/mL) as a standard. The degree of esterification was measured as well. The polysaccharide sample was saponified (0.5 M NaOH, 1 h) and after neutralization (1 M HCl), the amount of methanol released was determined using a combined enzyme-colorimetric method, using 4-amino-5-hydrazino-1,2,4-triazole-3-thiol (Purpald^®^) as a chromogen according to the previously reported method [32]. The qualitative estimation of rare sugars was performed according to the periodate-thiobarbituric acid colorimetric method of Karkhanis et al. [33] as described exhaustively by Ognyanov et al. [34].

#### 2.5.2. Determination of Molecular Weight

The determination of the average molecular weight of the EPS was performed using an Agilent 1220 high-performance size-exclusion chromatography-refraction index detector (HPSEC-RID) chromatography system, using pullulan standards with different molecular weights (0.59 × 10^4^–78.8 × 10^4^), a mobile phase of 150 mM NaH_2_PO_4_ (pH 7.0), and Agilent Bio SEC-3 column (300 A, 4.6 × 300 mm, 3 µm). The sample solution was diluted to a concentration of 1 mg/mL and filtered through a 0.45 μm membrane filter before analysis. Ten microliters of the sample were injected for analysis, and the retention time was recorded.

#### 2.5.3. Determination of Monosaccharide Composition

The monosaccharide composition of the EPS was determined using an HPLC-UV chromatographic system Agilent 1220 (Agilent Technologies, Waldbronn, Germany) employing the method of Honda et al. [35], with a modification by Yang et al. [36]. For this purpose, the EPS (1–5 mg) was hydrolyzed with 2.0 mL of 4M trifluoroacetic acid (TFA), in a sealed tube for 8 h at 110 °C. The released monosaccharides were derivatized with 1-phenyl-3-methyl-5-pyrazolone (PMP) to UV-absorbent products. Separation was performed using an Agilent TC-C18 column (5 μm, 4.6 × 250 mm) with a mobile phase 50 mM phosphate buffer (Na_2_HPO_4_-NaH_2_PO_4_, pH 6.9), with added acetonitrile, in gradient elution mode. Identification and quantification were based on response factors relative to standards subjected to the same hydrolytic procedure.

#### 2.5.4. FT-IR Spectroscopy

The Fourier transform infrared (FT-IR) spectra of EPS and its esterified derivatives were recorded at the absorbance mode by a Bruker IR-FT Spectrophotometer in a KBr tablet and analyzed in the wavelength range 500–4000 cm^−1^.

### 2.6. Anticancer Activity of Extracellular Polysaccharide/s

#### 2.6.1. Cell Viability Assay

The antiproliferative activity of EPS on human cervical carcinoma cells HeLa, mammary adenocarcinoma MCF-7, human keratinocyte HaCaT and mouse fibroblast BALB/3T3 cells was measured using 3-(4,5-dimethylthiazol-2-yl)-2,5-diphenyltetrazolium bromide (MTT) assay as previously described [37]. The assay detects the reduction of MTT by mitochondrial dehydrogenases to a blue formazan product, which reflects the normal function of mitochondria and cell viability. Briefly, the cells in exponential growth were plated at a final concentration of 1 × 10^4^ cells/well in a 96-well plate with 200 µL of growth medium. After overnight incubation, the growth medium was removed from each well and the cells were treated with rising concentrations of EPS (31.25 µg/mL, 62.5 µg/mL, 125 µg/mL, 250 µg/mL, 500 µg/mL, and 1000 µg/mL) and cultured in a humidified atmosphere with 5% CO_2_ for 24 and 48 h. Untreated cells were used as a negative control while cells treated with doxorubicin were used as a positive control. At the indicated times (24 and 48 h) the media was replaced with 100 μL of MTT solution (0.5 mg/mL) into each well. After incubation at 37 °C for 3 h, the medium was removed and 50 μL of DMSO/Ethanol (1:1 *v*:*v*) was added to each well to dissolve the purple crystals of formazan with shaking for 10 min. Absorbance was measured at 570 nm by a microplate reader (TECAN, SunriseTM, Grödig/Salzburg, Austria). The absorbance value of the control group (without treatment) was considered to be 100%. Relative cell viability was presented as a percentage relative to the control group based on the following formula: Cell viability = [OD of drug-treated sample/OD of non-treated sample] × 100. The 50% inhibitory concentration (IC_50_) value was determined as the concentration that caused 50% inhibition of cell proliferation. All experiments were performed in triplicate.

#### 2.6.2. Cell Migration Assay

The ability of EPS to inhibit the tumor cells’ migration was analyzed by performing a wound-healing scratch assay on HeLa cervical carcinoma cells. Cells were plated in 24-well plates at a density of 2.5 × 10^5^ cells/mL in DMEM containing 10% FBS, and they were cultured in a CO_2_ incubator at 37 °C, in an atmosphere with 95% humidity and 5% CO_2_, until a monolayer formed. A vertical cut was then created in each well of the plate using a sterile pipette tip with a 10 µL volume. The wells were washed twice with PBS to remove cell debris and were treated with EPS at a concentration of 250 µg/mL. Cell monolayers of untreated HeLa cells with the same vertical scratches served as a negative control of the experiments. The plates were cultured for 72 h in a CO_2_ incubator, and images of the scratched area were taken at regular time intervals (0, 24, 48, and 72 h) using an Olympus inverted light microscope with a digital camera to quantify the area of cell migration. The wound area in the control and treated cell cultures was measured at each time point using the ImageJ software package, Version 1.42.

#### 2.6.3. Fluorescence Microscopy

##### AO/EtBr Staining

Cell morphology changes after treatment with EPS were studied by staining the cells with a combination of the fluorescent DNA-binding dyes acridine orange (AO) and ethidium bromide (EtBr). For this purpose, cancer cells (1 × 10^5^ cells/well) were seeded on a cover slip placed on the bottom of each well in a 24-well plate and incubated overnight to form a monolayer on its surface. Then the cells were exposed for 24 h to the EPS at a concentration of 500 µg/mL, equal to half the maximal inhibitory concentration (IC_50_) established by the MTT assay. After an additional 24 h of incubation, the cells were stained with 10 µL of aqueous AO/EtBr solution (10 µL /mL of AO in PBS; 10 µL/mL of EtBr in PBS) for 5 min. Tumor cells cultured only in the medium were used as a negative control, and tumor cells cultured in the presence of doxorubicin were used as a positive control. The morphology changes in the AO/EtBr stained cells were observed under a fluorescent microscope (Leica DM 5000B, Wetzlar, Germany).

##### DAPI Staining

The nuclear morphology of EPS-treated HeLa tumor cells was also investigated by staining with the fluorescent dye 4’, 6 diamino-2phenylindole (DAPI). DAPI has a strong binding affinity for adenine-thymine-rich clusters. The DAPI molecule can pass through an intact cytoplasmic membrane, making it a suitable agent for studying the nuclear morphology of both live and fixed cells. Briefly, HeLa cells were seeded and treated with EPS as described in the previous section. Upon completion of the incubation, the cells were fixed with 3% paraformaldehyde for 10 min, washed three times with PBS, and stained with DAPI solution for 20 min at room temperature in the dark. Samples of treated and untreated (control) tumor cells were coated with Mowiol^®^, mounted on slides, and observed under a fluorescence microscope (Leica DM 5000B, Wetzlar, Germany).

##### Annexin V-FITC/PI Staining

Further, we assessed the apoptosis-inducing ability of EPS using an Annexin V-FITC Apoptosis Detection Kit: sc-4252 AK (Santa Cruz Biotechnology, Inc., USA). The translocation of phosphatidylserine from the inner to the outer side of the dual protein-lipid layer of the cell membrane is an early sign of apoptosis. HeLa cells were cultured on glass lamellae and treated with EPS, as described in the previous paragraphs. After that, the culture fluid was removed and the cells were coated with a solution of Annexin V-FITC and propidium iodide (PI), following the manufacturer’s instructions. The samples were incubated for 15 min at room temperature, in the dark, and then analyzed on a fluorescence microscope (Leica DM 5000B, Wetzlar, Germany) following a protocol specified by the manufacturer.

#### 2.6.4. Flow Cytometry

The effects of EPS on the cell cycle progression and apoptosis of HeLa cancer cells were analyzed by flow cytometry analysis. HeLa cells were grown to about 80% confluency and were then treated with the EPS at concentrations of 500 μg/mL. Untreated HeLa cells were used as controls. After 24 h of treatment, the medium was aspirated, the cells were washed twice with cold phosphate-buffered saline (PBS), trypsinized with Trypsin-EDTA (Sigma-Aldrich), and centrifuged at 1000 rpm for 10 min. Cell pellets were collected and processed for cell cycle and apoptosis analyses as described below.

##### Cell Cycle Analysis

The cell pellets of the control and EPS-treated HeLa cell cultures were washed with PBS, resuspended, and fixed with 70% ice-cold ethanol, which was added dropwise while vortexing. Cells were stored at -20ºC for at least 12 h. Before analysis, the fixed cells were washed with PBS, treated with RNase A (Roche Diagnostics GmbH, Mannheim, Germany) (20 µg/mL) for half an hour, and stained with propidium iodide (PI; 20 µg/mL). Cell populations at different stages of the cell cycle were analyzed by flow cytometer (Becton Dickinson, Mountain view, CA, USA). From each sample, 10,000 events were recorded, and the percentage of cells in different cell cycle phases (G1, S, and G2-M) was determined using FlowJo ™ v10.8 software (BD Biosciences, San Jose, CA, USA). Data are presented as the mean ± standard error of the mean of three replicates.

##### Annexin V/PI Analysis of Cancer Cell Apoptosis

Cells were stained using an Annexin V-FITC Apoptosis Detection Kit: sc-4252 AK (Santa Cruz Biotechnology, Inc. USA) according to the manufacturer’s protocol. After incubation for 15 min at room temperature, 10,000 cells from each sample were analyzed with a flow cytometer (BD FACSCalibur™) using the FlowJo software (BD Biosciences, San Jose, CA, USA).

#### 2.6.5. Immunocytochemical Analysis

The potential alterations in the expression and intracellular localization of the proliferation marker protein Ki67 and the apoptosis-related proteins p53 and bcl2 were investigated by immunocytochemical analysis of control untreated and EPS-treated HeLa cells. Cells were seeded on clean, sterilized cover slides with a density of 1 × 10^5^ cells/well in 24-well plates. After culturing for 24 h, a negative control (untreated cells), a positive control (cells treated with 2.25 µg/mL Dox), and cells treated with EPS at a concentration of 500 µg/mL were incubated for 24 h under the same conditions. The cover glasses were then washed with PBS, fixed with ice-cold methanol for 10 min, washed three times with PBS, and incubated overnight at 4C with p53 (Monoclonal rabbit Ab-5, clone DO-7, Thermo Scientific, Waltham, MA, USA), Bcl2 (Monoclonal rabbit Ab, clone E17, Zytomed Systems GmbH, Berlin, Germany), and Ki67 (Monoclonal rabbit Ab Clone SP148, Zytomed Systems GmbH, Berlin, Germany) antibodies. Visualization of the antigen-antibody reaction was performed using an immunocytochemical peroxidase reaction with polymer labeling and DAB chromogen (Novolink Polymer detection Systems RE7140-K, Leica Biosystems, Newcastle, UK) according to the manufacturer’s recommendations. The procedure was performed at room temperature and the slides were incubated in a humidity chamber. After staining, the preparations were washed several times with distilled water, stained with hematoxylin for 3 min, then dehydrated with an ascending series of alcohols and placed on slides using Bio-Mount DPX synthetic resin (Biognost, Zgreb, Croatia). The prepared immunocytochemical samples were observed with a Leica DM 5000B microscope, Leica Microsystems, Wetzlar, Germany, and documented with a Leica DFC 420 C camera, Wetzlar, Germany and Leica Suite 3.1.0 software.

### 2.7. Statistical Analysis

All experiments were performed in triplicate (n = 3) and the results were expressed as the mean ± SD (standard deviation). The significance of the differences between the control and experimental groups was analyzed using one-way analysis of the variables (ANOVA) followed by a post-hoc comparison test (Bonferroni) using GraphPAD PRISM software, Version 5 (GraphPad Software Inc., San Diego, CA, USA). A difference was considered statistically significant when the value of *p* < 0.05.

## 3. Results

### 3.1. Chemical Characterization of EPS Isolated from Coelastrella sp. BGV

In this study, we isolated and characterized a crude extracellular polysaccharide (EPS) extract from the green microalga Coelastrella sp. BGV, which is endemic to Varvara, Bulgaria. EPS was obtained through ethanol precipitation of a cell-free culture medium. The EPS was washed 3 times with 65% ethanol by centrifugation, dialyzed against distilled water, freeze-dried, and lyophilized. The freeze-dried EPS appeared as a pale yellow pliable fibrous polymer. It was analyzed for carbohydrate and protein content as well as neutral sugar and uronic acid content using colorimetric analyses. The chemical characteristics of EPSs are presented in Table 1. The EPSs contained mainly carbohydrates (36.4%), protein (7.14%), and small amounts of uronic acid (4.5%). The proteins detected in the EPSs were considered to have bonded with the polysaccharide molecules via O-glycosidic or N-glycosidic linkages.

**Figure 1:**
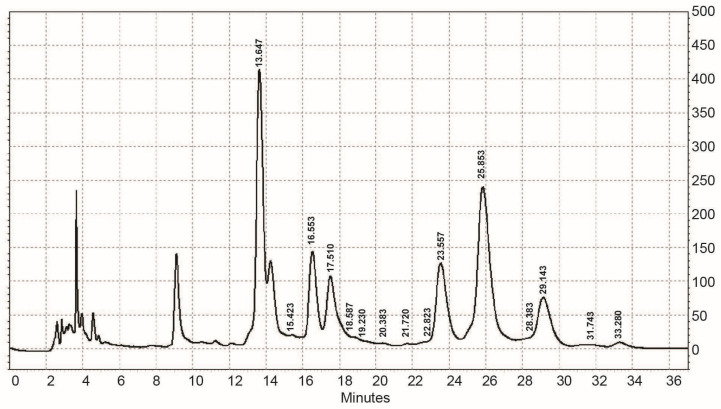
An HPLC-UV chromatogram of PMP (1-phenyl-3-methyl-5-pyrazolone)—monosaccharide derivatives obtained from EPS from *Coelastrella* sp. BGV.

The monosaccharide composition of EPS obtained from *Coelastrella* sp. BGV was determined by an Agilent 1220 HPLC-UV chromatography system after hydrolysis of the EPS and derivatization of the released monosaccharides to UV-absorbing products (Figure 1). It was established that EPS from *Coelastrella* sp. BGV is analyzed by employing seven different types of monomer unit standards. Two monosaccharides, including galactose and fucose, were identified, and five others could not be identified (Table 1). Interestingly, EPS tested strongly positive for rare sugars (a pink color was observed), and a positive test result with a 2-thiobarbituric acid reaction suggested the presence of not only 2-keto-3-deoxy-octonate, 3-deoxy-, and 3,6-dideoxy hexoses but also sialic acid and (N-acetyl)hexosamine. It is interesting to note that acetyl groups were not found, which suggests that there were not any acetylated sugar residues. Based on the analyses performed and the origin of the sample, it can be assumed that some of the unidentified monosaccharides are unacetylated galactosamine and/or glucosamine, or that the sample is a proteoglycan. The presence of anhydrous sugars (e.g., 3,6-anhydro-D/L-galactose) cannot be ruled out.

**Figure 2:**
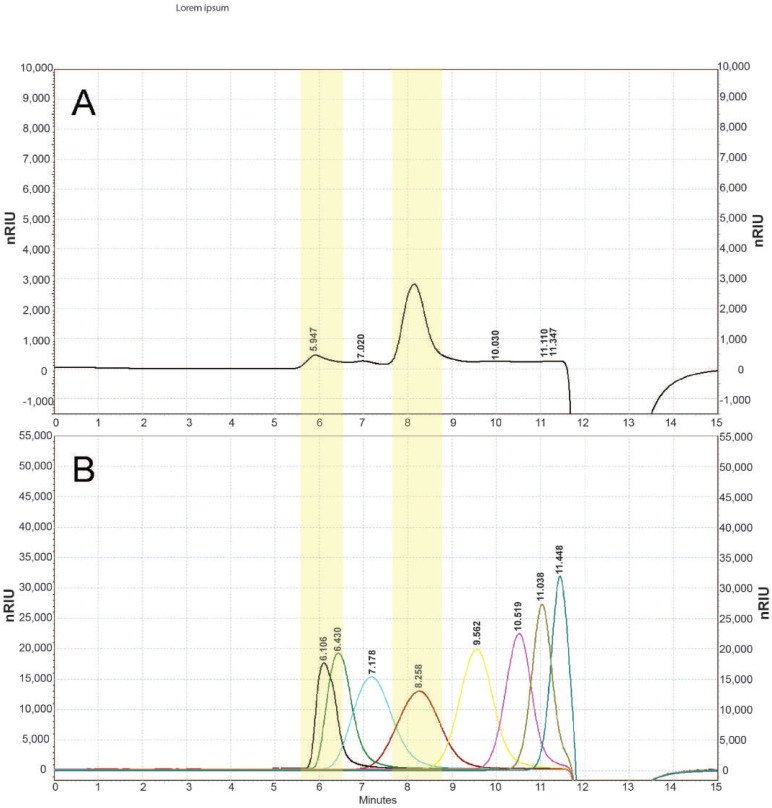
HPSEC elution profile of EPS from *Coelastrella* sp. BGV. (**A**) exopolysaccharide; (**B**) pullulan standards. Molecular weights of the standards used—from left to right: 78.8 × 10^4^, 40.4 × 10^4^, 21.2 × 10^4^, 11.2 × 10^4^, 4.73 × 10^4^, 2.28 × 10^4^, 1.18 × 10^4^, 0.59 × 10^4^ Da.

The molecular weight of the EPS obtained from *Coelastrella* sp. BGV was determined using the Agilent 1220 HPSEC-RID chromatography system. The MW of the EPS was calculated by the standard curve of the molecular weight (MW) over the retention time. The elution profile of the EPS is presented in Figure 2.

The molecular weight distribution of EPS showed the presence of three polysaccharide fractions (Figure 2A). The fraction with a molecular weight of 11.5 × 10^4^ Da (marked in the yellow zone of a pronounced peak) made up the largest amount (72.5% of total peak area). In front of it, two fractions (two small peaks) with a higher molecular weight, but in smaller quantities in the sample—72.4 × 10^4^ (11.8%) and 30.7 × 10^4^ (5.7%), respectively, were eluted. The data obtained show that the isolated EPS from *Coelastrella* sp. BGV has a medium molecular weight.

**Figure 3:**
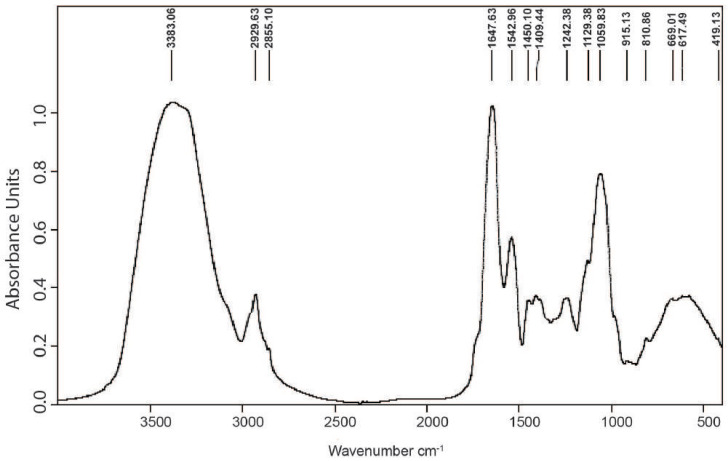
FT-IR absorption spectrum of the EPS from *Coelastrella* sp. BGV over the range of 4000–400 cm^−1^.

The IR-FT spectrum of the EPS on the IR-FT spectrophotometer in a KBr tablet was taken (Figure 3) and analyzed according to an available database. The individual absorption bands were compared with the presence of well-defined functional groups.

The IR spectrum showed a broad peak at 3400 cm^−1^ that corresponded to the stretching vibration of the hydroxyl groups (–OH), which were the characteristics of polysaccharides. The absorption observed at 3383 cm^−1^ was attributed to the stretching vibration of NH^3+^ (amine group); at 2929 cm^−1^ to the stretching vibration of the methylene (CH_2_) group (valence oscillations of the C–H bond of the CH_2_ group); at 1650–1550 cm^−1^ for the –NH_2_ group of the primary amine and the N–H group of the amide. The peak at 1409 cm^−1^ resulted from the stretching vibrations of the C-N bonds of the amines and amides (in the range of 1400–1418 cm^−1^). Bands characteristic of polysaccharide sulfate esters were not detected in the spectrum. The two bands noticed at 1542 and 1647 cm^−1^ were assigned to (N–H) and ν(C–N) of the amide II region and the N-linked C=O of the amide I structure vibrations. Bands pointed to the presence of amino sugars and/or protein components. The peak at 1535 cm^−1^ also corresponded to the monosaccharides’ C–OH bending vibration. Not found were absorption bands for acetyl groups, which confirmed previous findings made by spectrophotometric analysis. These findings demonstrated that the IR-FT spectrum of EPS from *Coelastrella* sp. BGV is typical of a polysaccharide (or proteoglycan) composed predominantly of neutral sugars.

### 3.2. Anticancer Activity of Extracellular Polysaccharide

#### 3.2.1. Effects of EPS from *Coelastrella* sp. BGV on Cell Viability

**Figure 4:**
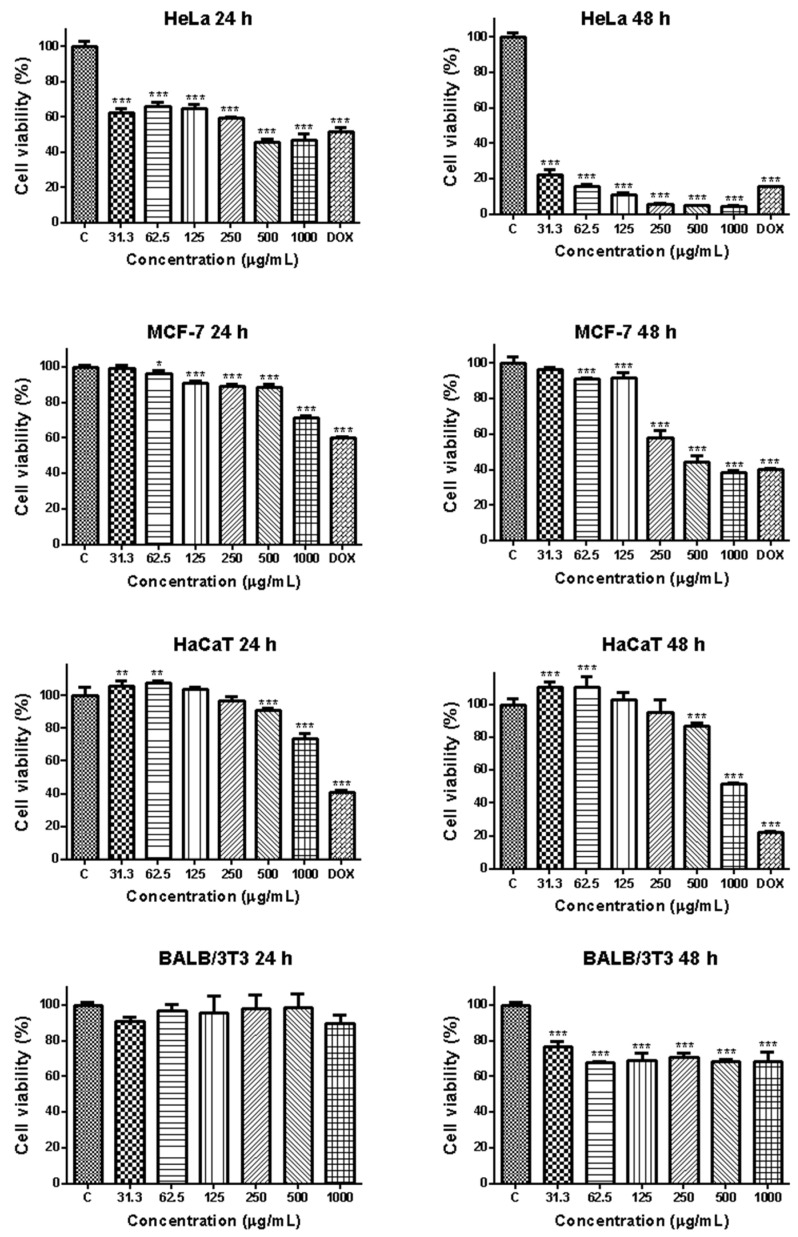
Effect of extracellular polysaccharide from Coelastrella sp. BGV on the viability of HeLa, MCF-7, HaCaT and BALB/3T3 cells assessed by an MTT test at 24 h and 48 h. Untreated cells and cultivated cells treated with the antitumor drug Doxorubicin (Dox; 2.5 μg/mL) were used as negative and positive controls, respectively. The data are expressed as the mean ± SD of five samples from each treatment group. * *p* < 0.05, ** *p* < 0.01, and *** *p* < 0.001 indicate significant differences compared to the negative control.

An MTT assay was used to determine the viability of the cancer cell lines HeLa and MCF-7 and the non-cancerous HaCaT and BALB/3T3 cells exposed to increasing concentrations (31.3, 62.5, 125, 250, 500, and 1000 μg/mL) of EPS for 24 and 48 h (Figure 4). The antitumor antibiotic doxorubicin (Dox), widely used in clinical practice for the treatment of human malignancies, was used as a positive control in the experiments. It was established that Dox significantly reduced HeLa cell viability/proliferation, with values between 5% and 54% at the 24th hour and between 1.0% and 17.0% at the 48th hour of treatment. The observed effect was concentration- and time-dependent. The IC_50_ values determined at the 24th and 48th hour were 2.254 μg/mL and 0.076 μg/mL, respectively.

As shown in Figure 4, EPS markedly inhibited the growth of tumor cells in a time- and concentration-dependent manner. It showed a moderate antiproliferative effect on HeLa cells; cell viability was between 45.77 ± 3.93% and 62.35 ± 4.78% at 24h with statistical significance compared to the control. At the high EPS concentrations—500 and 1000 μg/mL—cell viability was lower than that seen for the positive control (doxorubicin-treated HeLa cells). The antiproliferative effect of EPS on mammary carcinoma cells of the MCF-7 line was less pronounced, and the viability of the cells treated with the highest concentration was 71.57%. At the 48th hour, EPS caused a significant decrease in HeLa cell viability as compared to the control. Values were 3 to 10 times lower than those at 24 h and were 22.68 ± 4.296%, 16.04 ± 2.185%, and 11.15 ± 2.690% at EPS concentrations of 31.3 μg/mL, 62.5 μg/mL, and 125 μg/mL, respectively. The highest inhibitions of proliferation reached up to 6.033 ± 0.2418%, 4.878 ± 0.2218%, and 4.833 ± 0.4446% when EPS concentration increased to 250 μg/mL, 500 μg/mL, and 1000 μg/mL. The viability of MCF-7 cells also showed a marked time-dependent decrease, and values ranging between 96.4% and 38.6% were measured at the 48th h. Statistically significant reductions of cell viability compared to the untreated controls were found in all concentrations higher than 31.3 μg/mL.

The control non-cancer cell lines used in the study showed lower sensitivity to the cytotoxic effects of the EPS than the cancer cells. The viability of the human keratinocyte cells HaCa showed a statistically significant increase compared to the untreated control at the lowest tested concentrations of 31.3 μg/mL and 62.5 μg/mL. A decrease in cell viability was established only at the highest tested concentrations of 500 and 1000 μg/mL, with values of 91.17% and 73.96% for the 24th h and 86.77% and 51.75% for the 48th h.

The viability of the EPS-treated mouse fibroblasts BALB/3T3 was not decreased at 24 h and was equal to that of the control. At 48 h, the viability of the EPS-treated fibroblasts was between 76.74 ± 6.195% and 68.54 ± 2.899%. These values were about 10-fold higher than those of the EPS-treated tumor cells.

The inhibitory concentrations of EPS (IC_50_) were calculated by curve fit analysis of the obtained concentration-response curves for all cell lines tested. The results are presented in Table 2.

The results presented in Table 2 demonstrate a higher toxicity of EPS (lower IC_50_ values) for the cervical and breast carcinoma cell lines as compared to the non-cancer control cell lines. Based on the MTT results, the HeLa cell line was selected as the most suitable model system for the subsequent analyses of EPS’s anticancer potential.

#### 3.2.2. Effects of EPS on HeLa Cancer Cell Migration

**Figure 5:**
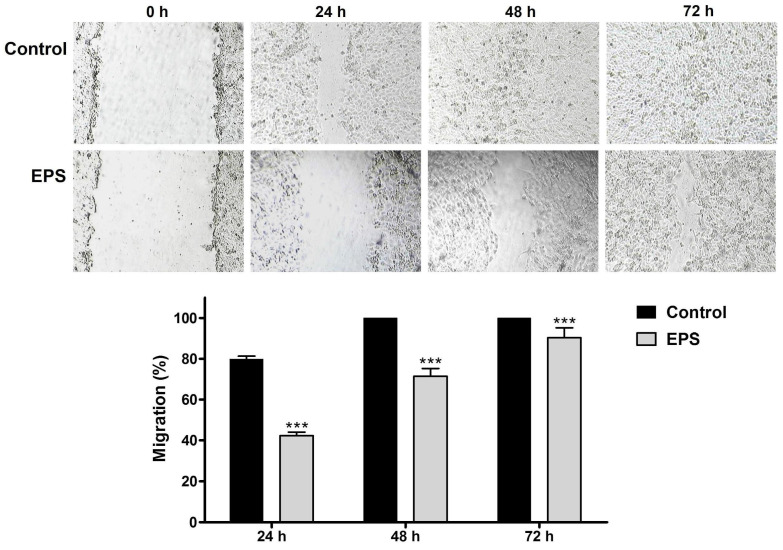
Effect of Coelastrella sp. BGV EPS treatment on the migration of HeLa cervical carcinoma cells evaluated by wound-healing assay. (**Upper panel**) Light microscopy images of untreated cell cultures and cell cultures treated with EPS (250 μg/mL); (**Lower panel**) Quantification of the EPS effect on the migration potential of the cancer cells. Data are presented as Mean ± SD; *** *p* < 0.001 compared to the untreated control.

The effect of EPS on the migration capacity of cervical carcinoma cells of the HeLa line was examined by a wound-healing assay. The EPS-induced changes in the migration ability of the cancer cells were followed in dynamics during the 72 h period, and measurements of the wound area were performed at regular time intervals (Figure 5).

In the control cell cultures, about 80% reduction of the wound width was measured at 24 h, and complete monolayer healing was observed as early as 48 h. The EPS treatment induced a statistically significant delay in the wound healing as compared to the control, and the mean percentages of cancer cell migration at the three time points measured (24, 48 and 72 h) were 42.3 ± 1.7%, 71.43 ± 3.8% and 90.4 ± 4.7%, respectively.

#### 3.2.3. Fluorescent Microscopy Analysis of EPS-Induced Apoptotic Alterations in HeLa Cancer Cells

**Figure 6:**
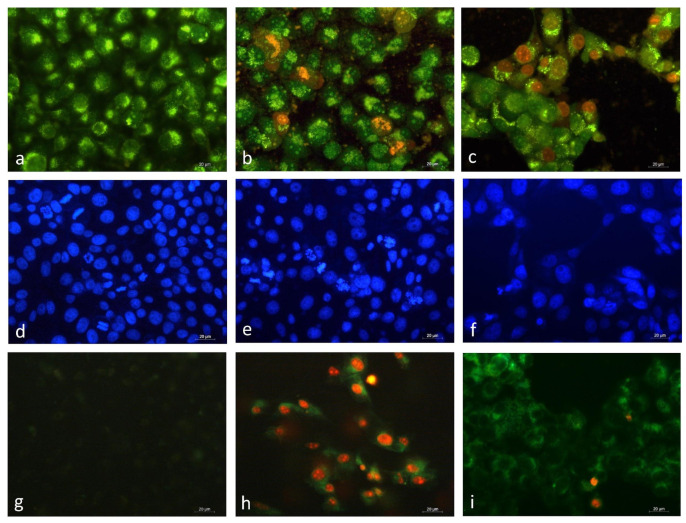
Morphological changes of the HeLa tumor cells cultured in the presence of EPS (500 μg/mL) for 24 h followed by AO/EB (**Upper row**), DAPI (**middle row),** and Annexin V (**Lower row**) staining. Fluorescence micrographs of: (**a**,**d**,**g**)—untreated HeLa cells; (**b**,**e**,**h**)—cells after incubation with 500 µg/mL of EPS; (**c**,**f**,**i**)—cells after incubation with 2.5 µg/mL Dox; Scale bar = 20 µm.

To determine whether the growth-inhibitory effect of EPS was related to the induction of apoptosis, HeLa cells were treated with an IC_50_ concentration of EPS for 24 h, and then their morphological changes were analyzed under a fluorescence microscope after double intravital staining with AO and EtBr (Figure 6). Acridine orange is a vital dye that stains both live and dead cells, whereas ethidium bromide stains only the cells that have lost their membrane integrity.

As shown in Figure 6, the HeLa control cells were morphologically normal, with nuclei of similar sizes, regularly shaped, and evenly bright green stain color (Figure 6a). The cells treated by EPS presented the typical morphological characteristics of early and late apoptosis. Wrinkled cells showing chromatin condensation as a bright green area or fragments (early apoptotic) and a significant number of cells with orange to red stained nuclei and condensed or fragmented chromatin (late apoptotic) were observed (Figure 6b). These alterations were accompanied by membrane blebbing and nuclear shrinkage. The nuclear morphology of the cells treated with Dox (Figure 6c) was completely destroyed. Late apoptotic cells with a bright red stained nucleus predominated. The majority of the HeLa cells were detached from the surface of the coverslips and were floating in the medium.

Further confirmation of the EPS-induced apoptosis of tumor cells was obtained by staining with DAPI. The control, untreated HeLa tumor cells had intact nuclei, round to slightly oval in shape and almost uniform in size, with smooth outlines and evenly distributed chromatin. Cell nuclei in different phases of mitosis were observed (Figure 6d). In contrast to the controls, the EPS-treated tumor cells showed significant morphological changes in the nuclei typical of apoptosis, such as nuclear polymorphism, chromatin condensation and margination, nuclear fragmentation, and collapse of the cell into membrane-bound apoptotic bodies (Figure 6e). The reduced number of nuclei with uneven outlines, condensed chromatin, and fragmented nuclei with multiple apoptotic bodies was seen in the samples treated with the anthracycline antitumor antibiotic doxorubicin (Figure 6f). The morphological studies showed that *Coelastrella* EPS was able to induce marked apoptotic morphology in HeLa cells at the cellular and nucleus levels.

The EPS-induced apoptosis of HeLa cells was also studied using Annexin-V staining, which permits the detection of phosphatidylserine exposure on the outer membrane surface of the apoptotic cells [38,39]. The translocation of phosphatidylserine from the inner side of the plasma membrane to the outer layer is one of the earliest steps in the initiation of programmed cell death-apoptosis. In this initial process, chromatin condensation occurs, but the cell membrane still retains its integrity. Annexin is a lipid-binding protein with an affinity for phosphatidylserine. Cells stained only with Annexin V-FITC glow green on the outside of the plasma membrane (early apoptotic). As cells move into late apoptosis, the permeability of the cell membrane is disrupted and DAPI enters the cell and stains the nuclei orange-red (late apoptotic). Late apoptotic cells were both Annexin V-FITC and PI positive. After EPS treatment, HeLa cells in the early and late apoptotic stages were observed under fluorescence microscopy, but those with late apoptosis (Annexin V-FITC and PI positive) predominated (Figure 6h). By contrast, after treatment with Dox, most cells were in the early apoptotic stage (Annexin V-FITC positive) (Figure 6i).

#### 3.2.4. Flow Cytometry Analysis of the Effects of EPS on the Apoptosis and Cell Cycle Progression in HeLa Cancer Cells

##### Apoptosis Assay

**Figure 7:**
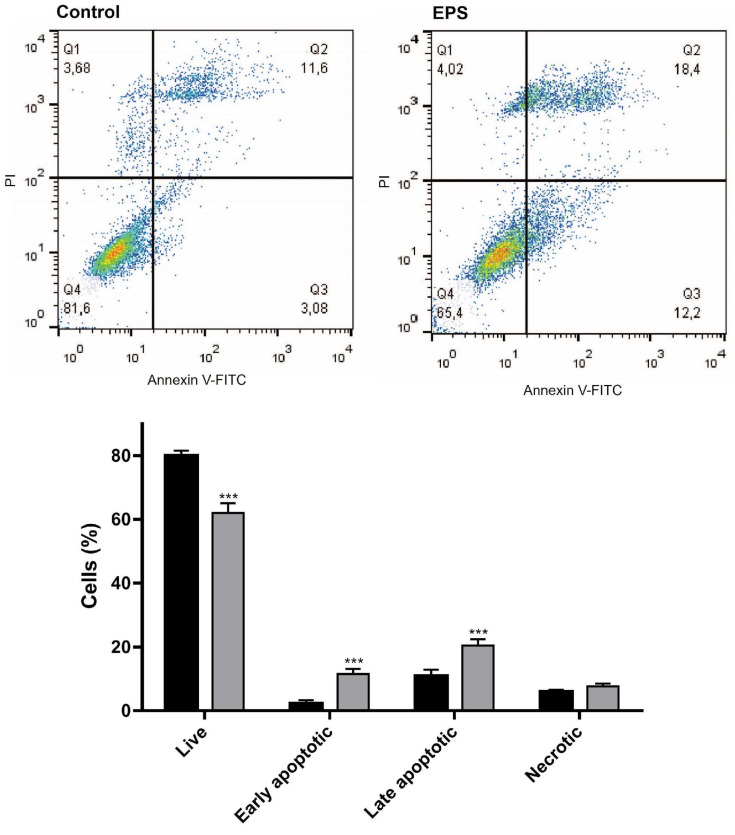
Proapoptotic effect of *Coelastrella* sp. BGV EPS on HeLa cervical carcinoma cells as evaluated by FACS analysis. (**Upper panel**) Representative histogram of control untreated cells and cells treated with 500 µg/mL EPS for 24 h. (**Lower panel**) Bar graph showing the percentages of the live, early and late apoptotic, and necrotic cells. The data are expressed as mean ± SD from three independent experiments; *** *p* < 0.001 indicates significant difference as compared to the negative control.

Quantification of the live, early and late apoptotic, and necrotic cells in the HeLa cell cultures treated with 500 μg/mL EPS for 24 h was performed by FACS analysis after Annexin V-FITC/PI fluorescent staining (Figure 7).

The performed Annexin V-FITC/PI flow cytometry demonstrated a statistically significant increase in the early and late apoptotic cells and a decrease of the percentage of live cells in the EPS-treated cell cultures as compared to the untreated control.

##### Cell Cycle Analysis

**Figure 8:**
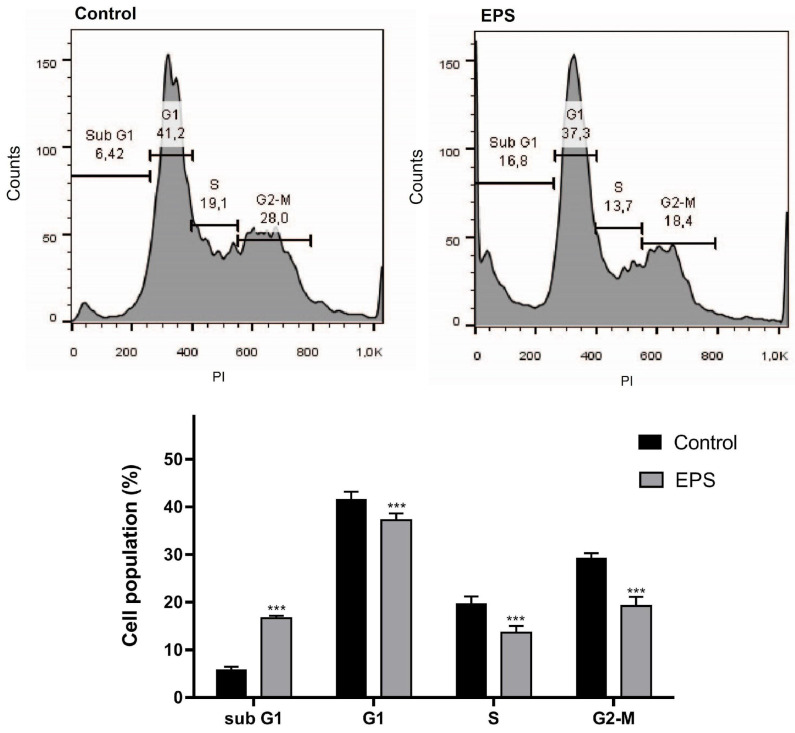
Effect of Coelastrella sp BGV EPS treatment on the cell cycle progression of HeLa cervical carcinoma cells. (**Upper panel**) Representative histogram of control untreated cells and cells treated with 500 µg/mL EPS for 24 h. (**Lower panel**) Bar graph representing the distribution of the cells in the different cell cycle phases. The data are expressed as mean ± SD from three independent experiments; *** *p* < 0.001 indicate significant difference as compared to the negative control.

The effect of EPS from *Coelastrella* sp. BGV on the cell cycle progression of HeLa carcinoma cells was studied by FACS analysis based on the detection of cellular DNA content after fluorescent staining with PI (Figure 8).

The results of flow cytometry analysis (presented in Figure 8) indicate that the EPS treatment induces a significant increase in the Sub G1 cell population and decreases the percentages of the G1, S, and G2-M cells.

#### 3.2.5. Immunocytochemical Analysis of the EPS-Induced Alterations in the Expression and Intracellular Localization of the p53, bcl2, and Ki67 Proteins

**Figure 9:**
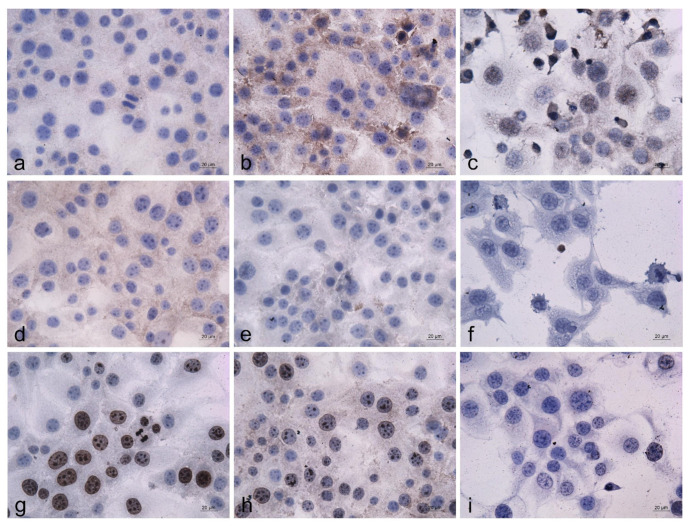
Immunocytochemical analysis of the EPS effects on the expression and intracellular localization of the p53, bcl2, and Ki67 proteins in HeLa carcinoma cells. (**a**,**d**,**g**) untreated control cells; (**b**,**e**,**h**) cells treated with EPS (500 μg/mL); (**c**,**f**,**i**) cells treated with Dox (2.5 μg/mL); (**a**–**c**) p53 immunostaining; (**d**–**f**) bcl2 immunostaining; (**g**–**i**) Ki67 immunostaining.

Immunocytochemical analysis of the HeLa cells treated with 500 μg/mL EPS for 24 h was performed to identify the potential alterations in the expression and intracellular localization of the tumor suppressor protein p53, proapoptotic protein bcl2, and proliferation marker protein Ki67. Untreated cells and cells treated with the standard cytostatic Dox were used as negative and positive controls, respectively (Figure 9).

The results of the performed analysis revealed marked alterations in the pattern and intensity of the immunochemical staining of the EPS-treated cancer cells. Nuclear p53 staining was not detected in the control and EPS-treated cells (Figure 9a,b). However, in the cells exposed to EPS, a prominent increase in the cytoplasmic staining intensity was observed (Figure 9b), suggesting the accumulation of p53 protein in the cellular cytoplasm. In contrast, some of the nuclei in the Dox-treated cell cultures were p53 positive (Figure 9c). The intensity of bcl2 cytoplasmic staining of both EPS- and Dox-treated cells (Figure 9e,f) was significantly decreased in comparison to the control (Figure 9d). The effect of the standard cytostatic Dox was more clearly expressed than those of the studied EPS. The immunostaining of the nuclear proliferation marker Ki67 revealed a significant reduction in the number of positive cells as well as a decrease in the staining intensity of the cancer cells after treatment with the studied EPS (Figure 9g,h). A similar but much more pronounced effect was detected in the cells treated with the anticancer drug Dox (Figure 9i).

## 4. Discussion

Despite the progress in cancer therapy, a number of negative factors such as the increasing incidence of the disease, limited efficacy, multidrug resistance, side effects, and others, stimulate efforts to develop new and effective treatment approaches, as well as to develop new drugs. Nowadays, medicinal substances from natural sources, which offer new and alternative options for cancer treatment, are gaining more and more popularity, compared to synthetic drugs [40]. The inhibition of cancer cell proliferation is a critical effect of anticancer agents. Another major target for cancer therapy is the induction of apoptosis, by which antitumor drugs kill cancer cells [41,42].

As a part of our efforts to explore the therapeutic potential of the local strains of microalgae, we undertook a systematic study on the green microalga *Coelastrella* sp. BGV, which is endemic to Varvara, Bulgaria. In this study, for the first time, we isolated EPS from the Bulgarian strain of green microalga and assessed its antitumor and apoptosis-inducing properties against cervical cancer cells of the HeLa line in vitro. The chemical composition analysis of EPS collected by ethanol precipitation of a cell-free culture medium showed that it was composed mainly of carbohydrates (36.4%) and contained small amounts of proteins (7,14%). The uronic acid content was 4.5%. Moreover, the molecular weight and monosaccharide composition of the isolated exopolysaccharide were also investigated. The presence of three polysaccharide fractions with molecular weights 11.5 × 10^4^ Da, 72.4 × 10^4^ Da, and 30.7 × 10^4^ Da were established by HPSEC-RID chromatography. HPLC-UV chromatography indicated that the isolated extracellular polysaccharides contain seven different types of monomeric units, two of which were identified as galactose and fucose. FT-IR absorption spectra confirmed the existence of various polysaccharide-characteristic substituent groups without establishing the presence of sulfate esters. The results showed that *Coelastrella* EPS is a typical polysaccharide (or proteoglycan) composed mainly of neutral sugars.

The different microalga and cyanobacteria species display significant variability in their ability to produce EPS as well as in the amount, structure, and chemical composition of the secreted EPS. A recent study compared ten species of microalgae that are of commercial interest as a functional food or nutritional supplement, in terms of biomass composition, cell-associated polysaccharides, EPS, and their chemical composition (monosaccharide profile, uronic acid content, and sulfates) [8]. EPSs were identified in only four species of microalgae (*Porphyridium cruentum*, *Odontella aurita*, *Arthrospira platensis,* and *Chlorella vulgaris*). The highest content of EPS was found in *P. cruentum*, a red microalga known for its high content of sulfated extracellular polysaccharides [43]. The most abundant monosaccharide in microalgal EPS is glucose, while fructose predominates in cyanobacterial EPS. In the EPS produced by *Rhodophyta*, xylose was found to predominate, followed by galactose, while in *Charophyta*, mainly uronic acids and fucose were detected. Galactose is the most abundant monosaccharide of EPS from *Chlorophyta*. Only a few studies reported EPS production and partial characterization in green alga species, for example *Dunaliella salina*, *Chlorella vulgaris*, *Chlorella elipsoidea*, and *Chlorella pyrenoidosa* [44,45,46,47]. Chemical composition analysis indicated that these EPSs consisted of different types of monosaccharides and their derivatives. The heteropolysaccharides identified in *Chlorella pyrenoidosa* were found to contain rhamnose, glucosamine, glucose, glucuronic acid, mannose, fucose, galactose, and xylose, and the EPSs from *Spirulina platensis* were composed mainly of glucose monosaccharides and rhamnose and smaller amounts of glucuronic acid, mannose, glucosamine, fucose, and xylose [48]. *Spirulina* sp. LEB 18 heteropolysaccharides were reported to contain glucose, galactose, xylose, glucuronic acid, rhamnose, fucose, arabinose, and galacturonic acid [49]. Polysaccharides isolated from *Chlorella pyrenoidosa* consist of two low molecular weight fractions (69,658 Da and 109,406 Da) that have the same qualitative and different quantitative monosaccharide profile. Both fractions contained rhamnose, mannose, glucose, and galactose and up to 10% unknown monosaccharides [50]. The dominant monosaccharide in one fraction was galactose (46.5%), and in the second rhamnose predominated (37.8%). From the extraction residue of *D. salina*, a crude polysaccharide extract (PD) containing four fractions—PD1, PD2, PD3, and PD4— and the two subfractions PD4a and PD4b were obtained [51]. The results of the monosaccharide analysis showed that PD1 and PD4a are acidic heteropolysaccharides containing glucose and galactose, respectively, and PD4a contains sulfated groups. PD2 and PD3 are glucans, while PD4b is a polysaccharide complex linked to nucleic acids by covalent bonds.

The quantity of the produced extracellular polysaccharides shows significant variations between the different species and strains of green microalga and is substantially impacted by cultivation conditions, the physiological state of the cells, culture age, and the extraction techniques and procedures used. Even EPS obtained from the same algal species by the same methods and conditions but from different lots can be distinguished [6,10,11,47]. The variations in the chemical structure, molecular weights, monosaccharide composition, and chain conformation determine the differences in the biological activities of the microalga-derived EPS.

The anticancer activity of the polysaccharides isolated from different microalga species has been frequently reported in recent years, and the potential mechanisms of action of polysaccharides have been investigated. The EPS derived from the microalga *Thraustochytriidae* sp. GA strain inhibited the proliferation of ovarian, breast, and colon cancer cell lines, altered cell cycle-related protein expression, and displayed immunomodulatory activity by inducing human B cell proliferation and altering T cell cytokine production [15]. The anticancer activities of polysaccharides derived from various species of the *Chlorella* genus in cell culture models have been reported. Polysaccharides from *Chlorella pyrenoidosa* inhibited the growth of A549 human lung cancer cells in vitro, in a dose-dependent manner [50]. Purified EPS from *Chlorella zofingiensis* and *Chlorella vulgaris* exhibited antitumor activities against human colon cancer HCT8 cells in vitro, with IC_50_ values of 1.70 and 3.14 mg/mL, respectively [47]. In another study, similar inhibitory effects of EPSs from *Chlorella pyrenoidosa*, *Scenedesmus* sp., and *Chlorococcum* sp. on the cell growth, proliferation, and colony formation of the human colon cancer cell lines HCT116 and HCT8 (with better inhibitory effects on HCT8 cells) were reported [52]. Aqueous EPS from *Graesiella* sp. demonstrated a dose-dependent antiproliferative effect on HepG2 (human hepatocellular carcinoma) and Caco-2 (human colon cancer) cell lines, with higher sensitivity reported for Caco2 cells [53].

Moreover, the anticancer efficacy of microalgal EPS has been confirmed by in vivo experiments. The two low molecular weight polysaccharides from *Pavlova viridis* were found to exert strong immunoenhancing activities (significantly increased lymphocyte and macrophage proliferation, and phagocytosis of macrophages), and to inhibit the in vivo growth of implanted S180 tumors in mice after intragastric administration [54]. EPS from *P. cruentum* increased the spreading and phagocytic ability of peritoneal macrophages, stimulated the proliferation of bone marrow cells in a dose-dependent manner, retarded tumor growth, and prolonged the survival time (by 10–16 days) of hamsters implanted with Graffi myeloid tumors [55]. Extracellular polysaccharides from the microalga *Crypthecodinium* sp. SUN suppressed the growth of lung adenocarcinoma tumors in nude mice without affecting their body weight [56].

Despite the numerous studies indicating the cancer-suppressive effects of algal EPSs, currently no data are available regarding the anticancer potential of EPSs isolated from *Coelastrella* species. In this study, we examined the effect of EPS on cell proliferation and the viability of HeLa and MCF-7 human cancer cells and the non-cancer control cell lines HaCaT and BALB/3T3. The cell proliferation of the EPS-treated cells was tested using an MTT assay after 24 and 48 h. The results showed that EPS decreased the proliferation of HeLa at 24 and 48 h in a time- and dose-dependent manner. The results also showed that EPS does not inhibit BALB/3T3 cell proliferation at 24 h, and even increases the proliferation of the human keratinocyte cell line HaCaT at the lower tested concentrations. A significant reduction in the cell viability of HaCaT cells was established only at a concentration of 1000 μg/mL at the 24 h and 500 μg/mL and 1000 μg/mL at the 48 h. The cell viability of BALB/3T3 cells treated for 48 h with EPS was significantly decreased as compared to untreated control. However, it was significantly higher compared to that measured for the tumor cells, thus indicating the EPS’s ability to differentially regulate the proliferation of tumor and normal cells. These results are consistent with the previously reported data demonstrating the anticancer potential of algal EPSs.

Several studies have demonstrated that the anticancer effects of microalga-derived EPS stem from their capacity to trigger apoptosis in cancer cells. A novel acid polysaccharide designated as XQZ3, with a molecular weight of 29.13 kDa and mainly composed of galactose and mannose extracted from *Chlorella pyrenoidosa,* led to the induction of mitochondrial dysfunction, autophagy, and apoptosis of cancer cells [57]. The sulfated polysaccharides obtained from *Tribonema* sp. and *Phaeodactylum tricornutum* significantly reduced the proliferation of the liver cancer cell line HepG2 by inducing cell apoptosis without affecting the cell cycle and mitosis of tumor cells, and it showed immune-modulatory activity by stimulating macrophage cytokine production (such as IL-6, IL-10, and TNF-α) [58,59]. EPS from *Gymnodinium* sp. A3 was cytotoxic against various human lymphoid cells, particularly MT-4 cells, and induced apoptosis in the cells, as demonstrated by morphological, flow cytometry, and DNA fragmentation investigations [60]. The same polysaccharide exhibited significant cytotoxicity toward human myeloid leukemia K562 cells by inducing apoptotic cell death through the inhibition of DNA topoisomerases I and II (two nuclear enzymes regulating apoptotic cell death) [61,62]. EPS produced by *Chlorella* sp. inhibited the proliferation and promoted the apoptosis of HeLa cervical cancer cells by activating the MAPK, TNF, and PI3K-Akt signaling pathways [63].

To obtain information about the processes and mechanisms underlying the cancer-suppressive effects of the *Coelastrella* EPS detected in the present study, additional cytomorphological, flow cytometric, and immunocytochemical analyses were performed. Based on the results obtained from the cell viability assays, the HeLa cell line was chosen as a model system for the further analyses because it showed the highest sensitivity to the EPS-induced antiproliferative and cytotoxic effects among the cell lines tested.

The effect of EPS on cancer cell migration was followed in dynamics for 72 h and quantified by the wound-healing assay that analyzes the ability of the cells to fill a gap mechanically created by scratching a cell monolayer. The surface of the wound area measured in the EPS-treated HeLa cells was notably larger and showed a statistically significant difference from those measured in the untreated control at all three tested time intervals, indicating the inhibitory action of EPS on cervical carcinoma cells’ migration.

Fluorescence microscopy examination of HeLa cervical cells treated with *Coelastrella* EPS and labeled with the fluorescent dyes AO/EB and DAPI was used to acquire information about the processes that mediate anticancer action and identify the type of cell death. Apoptotic cells are distinguished by the presence of specific cellular and nuclear morphology changes, which allows for their identification using cytomorphological approaches [64]. The observed membrane blebbing, chromatin condensation, nuclear fragmentation, and apoptotic body formation in the treated HeLa cells indicated the ability of the EPS to trigger apoptosis. The proapoptotic activity of the EPS was also confirmed by fluorescent microscopy after staining with Annexin V-FITC/PI. This method allows the detection of the translocation of phosphatidylserine from the inner to the outer membrane surface, which is an apoptosis-specific alteration in the lipid structure of the cytoplasmic membrane occurring at the earliest stage of the apoptotic process. The cytomorphological analysis performed by these three fluorescent methods showed consistent results and allowed the establishment of typical apoptotic alterations in EPS-treated HeLa cervical carcinoma cells.

A fluorescence-activated cell sorting (FACS) analysis of Annexin V-FITC/PI-stained HeLa cells was used to quantify the proapoptotic effects of *Coelastrella* sp. BGV EPS. The results of the investigation revealed a statistically significant increase in both early and late apoptotic cell counts in EPS-treated cell cultures as compared to the untreated control. This finding is in agreement with the results obtained by fluorescent microscopy analysis and provides further confirmation of the detected apoptosis-inducing ability of EPS.

To analyze the effect of EPS from *Coelastrella* sp. BGV on cell cycle progression of HeLa carcinoma cells, a FACS analysis of PI-stained cells was performed to quantify the cell populations in the different phases of the cell cycle based on the differences of their DNA content. The obtained results were consistent with the data of Annexin V-FITC/PI FACS analysis and indicated a significant increase of the cell population that had Sub G1 DNA content, which is an indication of the induction of apoptosis in EPS-treated cancer cells. The percentages of G1, S, and G2-M cell populations in the cell cultures treated with EPS were significantly decreased in comparison with the control, and this effect was most pronounced for the G2-M cells. That is an indication of the ability of EPS to reduce the mitotic activity of the cancer cell.

This finding is consistent with the results of the immunocytochemical analysis of the control untreated and EPS-treated HeLa carcinoma cells, demonstrating a marked decrease in the expression of the nuclear proliferation marker Ki67. Ki-67 is a nuclear protein highly expressed in cycling cells but significantly down-regulated in resting G_0_ cells and rapidly degraded upon cell cycle exit [65]. This feature has made Ki-67 a clinically important diagnostic and prognostic marker for grading the primary tumor and metastases and predicting the likelihood of relapses and survival rates [65]. The presented results clearly demonstrate the antiproliferative activity of the studied *Coelastrella* sp. BGV EPS.

The potential involvement of the apoptosis-related proteins p53 and bcl2 in the molecular mechanisms underlying the detected proapoptotic activity was also investigated by immunocytochemical analyses of the control untreated and EPS-treated HeLa carcinoma cells. The cellular protein p53 plays central roles in the regulation of cell cycle progression, DNA repair, and apoptosis [66]. Under normal conditions, the expression levels of p53 are very low. However, the exposure of the cell to internal and external stresses triggers a rapid increase in the intracellular p53 protein levels. Under stress conditions, the p53 protein governs cellular fate decisions and restricts the propagation of damaged cells. Functional loss of the p53 protein has been found in about half of human malignancies, highlighting its key role in cancer suppression. In addition to its known function as a nuclear transcription factor regulating the expression of a number of apoptosis-related genes, more recent studies have discovered that p53 has additional activities in the cytoplasm, where it triggers apoptosis through a transcription-independent mechanism by directly binding and inactivating the anti-apoptotic protein bcl2 [67]. The stress-induced accumulation of p53 in the cytosol is a hallmark of the transcription-independent pathway of p53-mediated apoptosis [68]. The increased intensity of the cytoplasmic p53 staining and the reduced bcl2 staining observed in the EPS-treated HeLa cells suggests that the activation of the cytosolic transcription-independent p53 pathway is a possible mechanism of the EPS-induced apoptosis.

## 5. Conclusions

The ability to distinguish cancer from non-cancerous cells, suppress tumor cell proliferation, and induce apoptosis in treated tumor cells is an important property of candidate anticancer drugs. The present study provided the first evidence that the EPS isolated from the *Coelastrella* sp. BGV can selectively inhibit the growth of human cancer cell lines in a dose-dependent and time-dependent manner. Moreover, the ability of the *Coelastrella* EPS to inhibit cancer cell proliferation and migration and to induce apoptosis was clearly demonstrated. The presented results reveal that the green microalga *Coelastrella* sp. BGV is a promising source of valuable bioactive compounds and provides a foundation for further investigations of the *Coelastrella* EPS as a potential anticancer drug in other cancer model systems.

## Figures and Tables

**Table 1:** Chemical characteristics of exopolysaccharide isolated from *Coelastrella* sp. BGV.

Biochemical Composition	Concentration, *w*/*w*%
Total protein content	7.1
Neutral sugars	
Rhamnose (Rha)	-
Arabinose (Ara)	-
Galactose (Gal)	1.7
Glucose (Glc)	-
Mannose (Man)	-
Xylose (Xyl)	-
Fucose (Fuc)	0.4
Total carbohydrates (Glc equivalent)	36.4
Total uronic acids content	4.5
Degree of methoxylation, mol%	0.74
Acetyl groups content	0.0
Rare sugars test (+ positive, − negative)	+++

**Table 2:** Inhibitory concentrations (IC_50_; μg/mL) of extracellular polysaccharides isolated from *Coelastrella* sp. BGV, determined by MTT test after 24 h and 48 h treatment of cancer and non-cancer cell lines.

Cell Line	24 h	48 h
HeLa	643.11	<31.3
MCF-7	>1000	486.40
HaCaT	>1000	>1000
BALB/3T3	>1000	>1000

## Data Availability

All data are comprised in the manuscript.
